# Association of *Hibiscus sabdariffa* and high-intensity interval training induces reduction in adiposity and beneficial metabolic adaptations in obesity without changes in lipid metabolism

**DOI:** 10.1590/1414-431X2024e13676

**Published:** 2024-11-04

**Authors:** D.B.O. de Oliveira, M.A. Giordani, R.A.M. Luvizotto, A.F. do Nascimento, M.C. dos Santos, K.C.C. Santos, A.P. Lima-Leopoldo, A.S. Leopoldo, M.M. Sugizaki

**Affiliations:** 1 Programa de Pós-Graduação em Educação Física, Centro de Educação Física e Esportes, Universidade Federal do Mato Grosso, Cuiabá, MT, Brasil; 2 Programa de Pós-Graduação em Ciências em Saúde (PPGCS), Universidade Federal do Mato Grosso, Campus Universitário de Sinop, Sinop, MT, Brasil; 3 Programa de Pós-Graduação em Ciências Fisiológicas, Centro de Ciências da Saúde, Universidade Federal do Espírito Santo, Vitória, ES, Brasil; 4 Programa de Pós-Graduação em Nutrição e Saúde, Centro de Ciências da Saúde, Universidade Federal do Espírito Santo, Vitória, ES, Brasil; 5 Programa de Pós-Graduação em Educação Física, Centro de Educação Física e Esportes, Universidade Federal do Espírito Santo, Vitória, ES, Brasil

**Keywords:** Obesity, Hibiscus sabdariffa, High-intensity interval training, Lipid metabolism

## Abstract

High-intensity interval training (HIIT) has stood out as a treatment for obesity, leading to adaptations of the cardiovascular system and reducing body adiposity. In addition, the search for alternative therapies for weight loss has intensified. The administration of *Hibiscus sabdariffa* (Hs) has been described as an efficient supplement in weight loss and in the treatment of metabolic changes associated with obesity. In this context, the objective was to investigate the effects of the association of Hs and HIIT on metabolic adaptations and lipid metabolism in obese rats. Wistars rats were subjected to obesity and subsequently randomized into 4 groups: obese (Ob), obese + HS (ObHs), obese + HIIT (ObHIIT), and obese + HS + HIIT (ObHsHIIT). For 8 weeks, ObHs and ObHsHIIT rats received *Hs* extract daily (150 mg/kg of body weight) and trained groups (ObHIIT and ObHsHIIT) were subjected to a HIIT program on a treadmill. Nutritional profile, glycemic curve, biochemical profile, and liver glycogen were determined. HIIT decreased caloric intake, feed efficiency, body adiposity, total body fat, and body weight gain, associated with improvements in physical performance parameters and a smaller glycemic curve and area. Hs had a hepatoprotective effect, reducing alkaline phosphatase values, but its effects were more pronounced when associated with HIIT. Therefore, the combination of treatments promoted a reduction in food consumption and body adiposity, as well as an improvement in physical performance and glycemic profile, but without changes in lipid metabolism.

## Introduction

Obesity is described as a chronic, low-grade inflammatory disease characterized by excessive accumulation of adipose tissue (1). It is currently considered a global public health problem, affecting developed and developing countries (1). According to the World Health Organization (WHO), in 2020, around 1.9 billion people in the world were overweight, and 650 million were obese (2). In this scenario, estimates for global overweight levels suggest that almost 3.3 billion adults could be affected by 2035, compared to 2.2 billion in 2020, among whom around 1 billion people will be obese (3). In Brazil, research data by telephone survey (Vigitel) about risks and protective factors for chronic diseases showed that 61.4% of the population was overweight in 2023, being 24.3% classified as obese (4).

The major problem of obesity is its association with increased risk of mortality, reduced life expectancy, and numerous comorbidities such as type 2 diabetes mellitus (DM), dyslipidemia, and respiratory, hepatic, digestive, chronic renal, and psychological diseases, cancers, and cardiovascular disorders (2). The energy imbalance that occurs in obesity leads to hypertrophy and hyperplasia of adipose tissue (5). However, excess calories can cause ectopic fat accumulation as adipose tissue cells reach their maximum storage capacity (5). In this way, triglycerides are stored in tissues such as the heart, liver, and muscles. However, both ectopic fat accumulation and adipose tissue expansion stimulate the secretion of pro-inflammatory adipokines such as leptin, interleukin-6 (IL-6), and tumor necrosis factor α (TNF-α) and inhibit the release of anti-inflammatory products such as adiponectin, thus modifying metabolic and inflammatory processes, and triggering metabolic changes such as hypertension, hyperinsulinemia, dyslipidemia, diabetes, and other conditions (6).

Given this context, it is important to search for interventions that aim to reverse obesogenic factors. Among the suggestions are dietary re-education, reducing the intake of high-calorie foods, fats, and simple sugars, and implementing the consumption of healthy foods, in addition to practicing regular physical activity (2). High-intensity interval training (HIIT) is recognized as an effective exercise approach in which periods (30 s to several minutes) of high-intensity effort (≥80%, but often 85-95% of maximal heart rate and recovery) are alternated with 1 to 5 min of recovery (rest or low-intensity exercise for recovery) (7). This methodology induces significant metabolic stress, leading to physiological adaptations that improve cardiorespiratory fitness, aerobic and anaerobic capacity, and calorie expenditure during and after exercise (7). Studies show that HIIT contributes to reducing body fat by increasing total calorie expenditure and promoting fat burning. It also has numerous benefits, including a reduction in circulating glycemia and an improvement in biomarkers associated with glucose metabolism, thus preventing progression to DM in obese individuals (7-10). Furthermore, the reduction in total and low-density lipoprotein (LDL) cholesterol, and the increase in high-density lipoprotein (HDL) concentrations caused by HIIT help control blood pressure and lipid profile, reducing the risk of cardiovascular diseases (11).

In contrast to traditional continuous aerobic exercise, HIIT has demonstrated comparable or superior effects on visceral and hepatic fat loss and improvement on ventricular and endothelial function and peak rate of oxygen consumption (VO_2_peak) (7,10). For DM and hypertension, HIIT has shown to be equivalent to or better than moderate-intensity continuous training (MICT) in reducing insulin resistance (9) and blood pressure (10). Moreover, HIIT has the advantage of being more time-efficient, making it a promising non-pharmacological strategy to improve overall health and manage obesity-related risks (7,8).

In addition to the practice of physical training, other adjuvant tools, such as natural products and foods with bioactive properties, are being described as alternative therapies for obesity and its comorbidities. In this context, studies with *Hibiscus sabdariffa* (Hs), a botanical species belonging to the Malvaceae family, showed that this plant can be an important tool in the non-pharmacological adjuvant treatment of obesity and associated comorbidities (12).

Research has associated Hs with the lowering of blood pressure, improved lipid profile, and cardiovascular protection (13-15). A study demonstrated that Hs treatment in rats with metabolic syndrome caused a reduction in body mass and intra-abdominal fat, as well as in triglycerides, insulin, and leptin levels in relation to the group with metabolic syndrome without treatment (16). The authors also observed that HS treatment can preserve renal structure, increase antioxidant markers, and decrease lipid peroxidation in the kidneys (16). Prasomthong et al. have demonstrated that HS treatment improved antioxidant properties and attenuated hepatic steatosis, liver inflammation, oxidative stress, and insulin resistance in rats fed a high fat diet (17). A previous study by our group showed that in obese rats, the group supplemented with Hs presented lower final body mass, body mass gain, and body adiposity index (AI) than the untreated group (18). Thus, scientific evidence points to the therapeutic effects of the use of Hs in the treatment of many diseases and obesity, especially taking into account the improvement in body fat and prevention of associated morbidities such as diabetes, hypertension, and dyslipidemia.

Therefore, considering the lack of studies that demonstrate the effect of HIIT in combination with the use of plants with medicinal properties, the objective of this study was to investigate the effect of the administration of Hs extract combined with HIIT and its protective role in metabolic and lipid changes induced by obesity. The hypothesis was that HIIT alone or associated with Hs reduces adiposity and promotes positive effects on metabolic parameters and hepatic metabolism.

## Material and Methods

### Animal care and experimental design

Thirty-seven Wistar rats, 60 days old, from the Central Animal Facility of the Federal University of Cuiabá (UFMT), Brazil, were used. Initially, the animals were distributed and randomized into 2 groups according to the type of diet received: standard diet (Control group; C: n=8) and high-fat diet (Obese group; Ob: n=29). Initial body weight (IBW) was similar between groups (C: 272±42 g *vs* Ob: 259±36 g; P>0.05). After eight weeks of obesity induction, the Ob animals were redistributed into 4 groups according to HIIT and Hs protocols for 8 weeks: obese (Ob, n=8), obese + *Hibiscus sabdariffa* (ObHs, n=8), obese + HIIT (ObHIIT, n=6), and obese + *Hibiscus sabdariffa* + HIIT (ObHsHIIT, n=7). Throughout the period, body weight was measured weekly and food consumption, caloric intake, and feed efficiency were monitored daily.

The rodents were kept in collective cages at an ambient temperature (24±2°C) and controlled humidity (55±5%) in an inverted 12-h light/dark cycle. The study protocol was approved by the UFMT Ethics Committee under number 23108.722977/2017-35 and followed recommendations from the Guide for the Care and Use of Experimental Animals and the Ethical Principles in Animal Experimentation of the Brazilian College of Animal Experimentation (COBEA).

### Experimental diet

Rats in the control diet group (C) received normocaloric rodent chow (NUVILAB CR-1, Brazil) and water *ad libitum*. The Ob, ObHIIT, ObHs, and ObHsHIIT groups received a high-fat diet from NUVILAB CR-1 food powder, casein, lard, condensed milk, cornstarch biscuits, vitamin and mineral mixture, and water with sucrose (300 g/500 mL), as demonstrated in Table 1.

**Table 1:** Composition of experimental diets.

Components	Diets	
	Standard diet	High-fat diet*
Carbohydrate	65.6	45.2
Protein (casein >99%)	22	20.9
Fat	4	24.5
Fibers	4	4
Vitamin mix^+^	1	Added
Mineral Blend^+^	3.5	Added
Total (%)	100	100
Energy (kcal/100 g)	380	485^#^
Energy (kj/100 g)	1.591	2.031^#^

The NUVILAB CR-1 commercial diet contains fat from soybean oil and carbohydrates as a sum of starch and sucrose. *The high-fat diet prepared by our group contained commercial powdered food, casein, corn biscuits, condensed milk, lard (main source of lipids), vitamins, and minerals (4.5%). ^+^Based on the amounts of vitamins/minerals in the diet, for every 100 g of the high-fat diet the following was added: iron: 25.2 mg; potassium: 104.8 mg; selenium 73.1 µg; molybdenum sulfate: 150.0 µg; vitamin B12: 34.5 µg; vitamin B6: 6 mg; biotin: 0.12 mg; vitamin E: 48.9 IU; vitamin D: 2.447.0 IU; and vitamin A: 15.291.2. ^#^Calories from sugar in drinking water (1.2 kcal/mL) are not included.

### Preparation and administration of *Hibiscus sabdariffa*

Hs was purchased from Chá e Cia store (www.chaecia.com.br). The hibiscus powder was diluted in absolute ethyl alcohol in a 10 g:100 mL ratio. This solution was stored in the refrigerator for seven days and stirred for 2 h daily. After this period, the solution was filtered, followed by alcohol evaporation in a rotary evaporator. The extract was then dried in an oven for two days at 37°C, and subsequently stored and diluted in distilled water. The rats received Hs extract daily (150 mg/kg of body weight) for 8 weeks.

### HIIT protocol and maximum progressive exercise test

The physical training protocol was adapted from Kemi et al. (19) as it has been proposed as a suitable strategy to promote beneficial cardiorespiratory fitness. In this context, trained groups (ObHIIT and ObHsHIIT) were subjected to a HIIT program on a treadmill (8 min at a speed corresponding to 80% of maximum speed and 2 min at a speed corresponding to 20% of maximum speed for 8 weeks, with a daily training time of 60 min) (20).

ObHIIT and ObHsHIIT animals were subjected to the maximum progressive effort test (MPET) on a motorized treadmill at the beginning and end of the HIIT training protocol, adapted from a previous protocol (19,20). The test was used to evaluate their pre/post-training physical performance and to determine the training load based on the maximum speed, duration, and distance covered. Thus, the rats were placed on a treadmill at an initial speed of 10 m/min, which progressively increased (2 m/min) every 2 min until they were exhausted. The exhaustion criterion adopted was the inability to maintain the required speed for 5 s. In this way, data on maximum speed, distance, and time were collected during the test.

In addition, to evaluate the intensity of the effort, the blood lactate concentration was determined immediately after MPET. A portable lactimeter (Roche, Accutrend Plus, Germany), capable of quantifying the lactate concentration in small amounts of blood, was used. For each test, a drop of blood was collected from the end of the animal's tail.

### Glucose tolerance test

Blood samples were collected from the caudal artery of the animals at baseline after a 6-h fasting period. Afterwards, a glucose solution (2.5 g/kg of body mass) was administered via orogastric gavage (21), and blood samples were collected at baseline, 15, 30, 60, 90, and 120 min. Blood glucose levels were measured using a portable glucometer (Accu-Chek Go Kit, USA). To evaluate glucose tolerance, the curve profile and area under the glycemic curve was determined by trapezoidal approximation of glucose levels. Glucose levels at x min were defined as glucose (x), and the area under the curve (AUC) for glucose (mg/dL/min) was calculated as previously described (21).

### Euthanasia

At the end of the experimental protocol, after fasting for 8 h, the animals were injected with sodium heparin (1000 U/kg; *ip*), then anesthetized and sedated with ketamine (70 mg/kg; *ip*) and xylazine (10 mg/kg; *ip*). After euthanasia, the animals underwent median thoracotomy for collection of blood and tissue samples.

### Nutritional and overall profiles

The AI is considered an important indicator of obesity used to evaluate the amount of body fat in animals (22). To this end, after euthanasia, the epididymal, visceral, and retroperitoneal fat deposits of the rats were dissected and weighed. The AI was calculated by the sum of fat deposits (total body fat), normalized by the animal's body weight [epididymal + retroperitoneal + visceral)/body weight × 100] (22).

### Lipid profile and liver glycogen

After euthanasia, liver tissue samples were collected and stored in a freezer at -80°C. Serum concentrations of glycogen, triacylglycerol, total cholesterol, high- (HDL) and low-density (LDL) lipoproteins, creatinine, albumin, urea, aspartate aminotransferase (AST), and alkaline phosphatase (ALT) were determined in liver tissue using commercial kits (CELM^®^, Brazil) and analyzed by the automated enzymatic colorimetric method.

### Statistical analysis

Statistical analysis was conducted in the GraphPad Prism 8 program (USA). Data are reported as means±SD and were submitted to the Shapiro-Wilk test to verify adherence to normality distribution. Analyses between control and obese groups were performed using the Student's *t*-test (comparison between two independent groups). Nutritional and overall profiles, adiposity, morphological investigation, physical performance, and lactate analysis in obese groups was performed by two-way analysis of variance (ANOVA) for independent samples (comparison between four groups, considering physical exercise and administration of *Hibiscus sabdariffa* as factors) and complemented with Tukey's *post-hoc* test. The glucose tolerance test was submitted to two-way repeated measures ANOVA for independent samples, complemented with Tukey's multiple comparison test. Effect size (Cohen's d) was used to describe the standardized mean difference of an effect (obesity) and partial eta squared for main effects (HIIT and Hs factors) was calculated from the ANOVA (η^2^p). The significance level considered for all variables was 5%.

## Results

Table 2 shows the nutritional and overall profiles, as well as the adiposity data of the experimental groups. Regarding the dietary profile, a statistical difference was observed between groups C and Ob, in which group C presented significantly higher values of food consumption (P=0.03, effect size 5.66) compared to Ob, without alterations in caloric intake (P=0.13, effect size 2.48). Nevertheless, the feed efficiency was elevated in Ob in relation to C (P=0.007, effect size 11.88). Considering the treated groups, the results indicated that the ObHIIT (F_1,4_=26.24, P=0.007, η^2^_P_=0.87) and ObHsHIIT groups (F_1,4_=6.948, P=0.01, η^2^_P=_0.06) presented reduced caloric intake (a reduction of 31 and 26.3%, respectively) compared to Ob. In addition, the feed efficiency was higher in Ob than ObHs (F_1,4_=2.273, P=0.21, η^2^_P=_0.36), ObHIIT (F_1,4_=106.8, P=0.0005, η^2^_P_=0.96), and ObHsHIIT groups (F_1,4_=24.47, P=0.008, η^2^_P_=0.86), respectively (increase of 32.3, 136, and 80.6%, respectively). The food consumption was also reduced in ObHIIT in relation to Ob (F_1,4_=21.44, P=0.01, η^2^_P_=0.84).

**Table 2:** Nutritional profile and adiposity of experimental groups during treatment.

Variables	Groups				
	C	Ob	ObHs	ObHIIT	ObHsHIIT
FC (g/day)	110±8	67.7±6.9*	56.8±3.4	46.2±3.3^#^	50.8±0.4
CI (kcal/day)	418±29	341±33	285±15	235±12^#^	251±5.5^#^
FE (%)	1.21±0.08	6.63±0.64*	5.01±0.26^#^	2.81±0.15^#^	3.67±0.08^#^
IBW (g)	435±62	517±55*	466±106	478±40	485±51
FBW (g)	475±72	697±79*	581±134	531±66^#^	559±87
BW gain (g)	40±20	180±44*	114±23^#^	52±32^#&^	74±37^#^
Epididymal (g)	8.9±4.8	23±4*	19±6	14±3^#^	13±5^#^
Visceral (g)	6.1±4.0	26±7*	17±10	16±4	13±7^#^
Retroperitoneal (g)	9.5±4.0	37±10*	31±11	23±3^#^	19±6^#^
Total body fat (g)	25±12	85±18*	67±26	52±8^#^	46±18^#^
AI (%)	5.0±1.8	12±1*	11±2	10±1	8.0±2.0^#&^

FC: food consumption; CI: caloric intake; FE: feed efficiency; IBW: initial body weight; FBW: final body weight; BW: body weight, AI: adiposity index. Groups C: control (n=8); Ob: obese (n=8); ObHs: obese + *Hibiscus sabdariffa* (n=8); ObHIIT: obese + high-intensity interval training (n=6); ObHsHIIT: obese + *Hibiscus sabdariffa* + high-intensity interval training (n=7). Data are reported as means±SD. Student’s *t*-test for independent samples (C and Ob groups). *P<0.05 C *vs* Ob; Two-way ANOVA for independent samples in obese groups (Ob, ObHS, ObHIIT, and ObHsHITT), complemented with Tukey's *post hoc* test. ^#^P<0.05 other groups *vs* Ob; ^&^P<0.05 *vs* ObHs.

In relation to nutritional profile, adiposity (Table 2), a statistical difference was observed between groups C and Ob regarding final body weight (FBW; P<0.0001, effect size 2.94), body weight gain (P<0.0001, effect size 4.096), epididymal (P<0.0001, effect size 3.19), visceral (P<0.0001, effect size 3.49), and retroperitoneal fat pads (P<0.0001, effect size 3.61), total body fat (P<0.0001, effect size 3.92), and AI (P<0.0001, effect size 4.81), respectively (Ob>C). Concerning treated groups, the body weight was reduced in ObHs (F_1,25_=1.473, P=0.24, η^2^_P_=0.056), ObHIIT (F_1,25_=6.754, P=0.02, η^2^_P_=0.21), and ObHsHIIT (F_1,25_=4.013, P=0.06, η^2^_P_=0.14) compared to the Ob group. In addition, the results showed that there were no differences between Ob and ObHs groups for the other parameters. In relation to the effects of HIIT, FBW (F_1,25_=6.754, P=0.02, η^2^_P_=0.21), body weight gain (F_1,25_=35.43, P<0.0001, η^2^_P_=0.58), epididymal (F_1,25_=15.27, P=0.001, η^2^_P_=0.38) and retroperitoneal (F_1,25_=16.25, P=0.0005, η^2^_P_=0.39) pads fats, as well as total body fat (F_1,25_=13.95, P=0.001, η^2^_P_=0.36) were reduced in ObHIIT compared to the Ob group. In this sense, HIIT protocol promoted a reduction of 39 and 38% in epididymal and retroperitoneal pads, respectively, as well as in total body fat and obesity (39%). With respect to the isolated effect of HIIT, the results indicated that this approach promoted a reduction of 27.3% in adiposity index (ObHsHIIT *vs* ObHs; F_1,25_=16.17, P=0.0005, η^2^_P_=0.39). Regarding the effect of Hs and the Hs plus HIIT interaction, a statistical difference was observed between the ObHIIT and ObHsHIIT groups for body weight gain (ObHsHIIT > ObHIIT, F_1,25_=9.45, P=0.005, η^2^_P_=0.27).

Table 3 shows the data from the morphological analysis of the experimental groups. A statistical difference was observed between the C and Ob groups, in which the Ob group presented the highest values for liver (P=0.0006, effect size 2.31), heart (P<0.0001, effect size 3.09), and heart/tibia ratio (P<0.0001, effect size 4.0) (Ob>C). No effect of Hs (ObHIIT *vs* ObHsHIIT) and HIIT (ObHs *vs* ObHsHIIT) on macroscopic analyses was observed.

**Table 3:** Morphological analysis of the groups.

Variables	Groups				
	C	Ob	ObHs	ObHIIT	ObHsHIIT
Gastrocnemius (g)	2.44±0.56	2.81±0.21	2.65±0.70	2.76±0.28	2.98±0.34
EDL (g)	0.19±0.04	0.21±0.02	0.20±0.03	0.21±0.02	0.22±0.02
Liver (g)	12.7±2.0	18.2±2.7*	14.7±3.0	15.2±1.9	14.6±2.6
Heart (g)	1.27±0.12	1.61±0.10*	1.36±0.17	1.34±0.10	1.43±0.25
Tibia length (cm)	4.38±0.17	4.38±0.11	4.30±0.15	4.27±0.06	4.36±0.08
Gastrocnemius/tibia length (g/cm)	0.55±0.12	0.64±0.04	0.62±0.16	0.64±0.06	0.68±0.07
EDL/tibia length (g/cm)	0.04±0.01	0.05±0.004	0.05±0.01	0.05±0.005	0.05±0.005
Heart/tibia length (g/cm)	0.29±0.02	0.37±0.02*	0.32±0.03	0.31±0.02	0.33±0.06

EDL: extensor digitorum longus. Groups C: control (n=8); Ob: obese (n=8); ObHs: obese + *Hibiscus sabdariffa* (n=8); ObHIIT: obese + high-intensity interval training (n=6); ObHsHIIT: obese + *Hibiscus sabdariffa* + high-intensity interval training (n=7). Data are reported as means±SD. Student *t*-test for independent sample (C and Ob groups). *P<0.05 C *vs* Ob; Two-way ANOVA for independent samples in obese groups (Ob, ObHS, ObHIIT, and ObHsHITT), complemented with Tukey's *post hoc* test.

**Figure 1:**
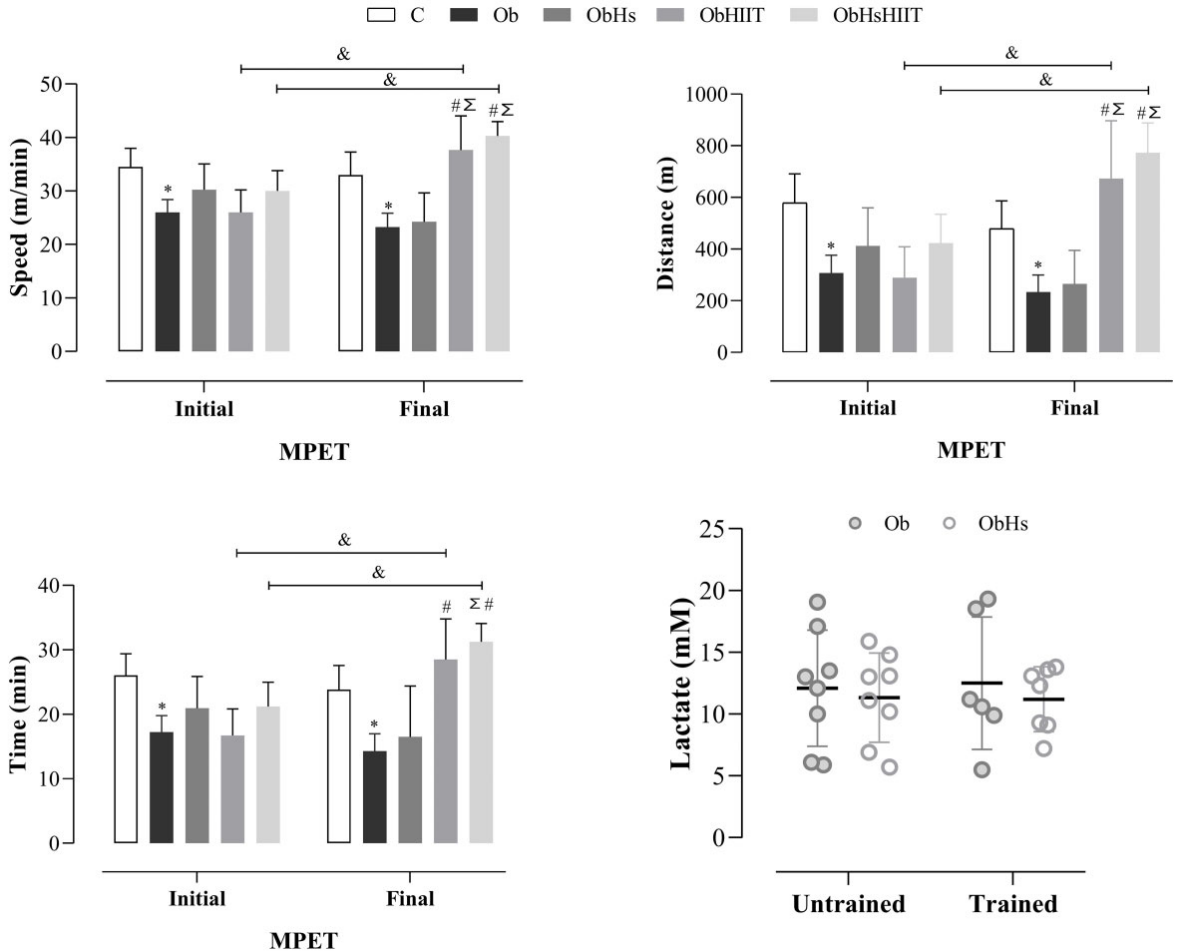
Effect of the interaction of *Hibiscus sabdariffa* and high-intensity interval training on physical performance and lactate analysis of the experimental groups. Maximum progressive effort test (MPET). Groups C: control (n=8); Ob: obese (n=8); ObHs: obese + *Hibiscus sabdariffa* (n=8); ObHIIT: obese + high-intensity interval training (n=6); ObHsHIIT: obese + *Hibiscus sabdariffa* + high-intensity interval training (n=7). Data are reported as means±SD. Two-way ANOVA for independent samples, complemented with Tukey's *post hoc* test. *P<0.05 Ob *vs* C; ^#^P<0.05 other groups *vs* Ob; ^Σ^P<0.05 other groups *vs* ObHs; ^&^P<0.05 Initial MPET *vs* Final MPET.

Figure 1 shows the data of the initial and final MPET carried out during HIIT training and lactate analysis. A statistical difference was observed between groups C and Ob regarding speed (Obesity effect: F_1,28_=61.86, P<0.0001, η^2^_P_=0.69; Test effect: F_1,28_=3.35, P=0.078, η^2^_P_=0.10; and Obesity × Test effect: F_1,28_=3.13, P=0.59, η^2^_P_=0.01), distance (Obesity effect: F_1,28_=64.82, P<0.0001, η^2^_P_=0.70; Test effect: F_1,28_=7.18, P=0.012, η^2^_P_=0.20; and Obesity × Test effect: F_1,28_=0.178, P=0.67, η^2^_P_=0.006), and time (Obesity effect: F_1,28_=69.28, P<0.0001, η^2^_P_=0.71; Test effect: F_1,28_=5.45, P=0.03, η^2^_P_=0.16; and Obesity × Test effect: F_1,28_=0.104, P=0.75, η^2^_P_=0.004) parameters in the initial and final MPET, respectively (C>Ob). In relation to speed, distance, and time parameters, the ObHIIT (speed: F_1,25_=83.24, P<0.001, η^2^_P_=0.77; distance: F_1,25_=83.69, P<0.0001, η^2^_P_=0.77; and time: F_1,25_=43.44, P<0.0001, η^2^_P_=0.63) and ObHsHIIT (speed: F_1,25_=0.23, P=0.63, η^2^_P_=0.009; distance: F_1,25_=0.44, P=0.51, η^2^_P_=0.02; and time: F_1,25_=0.01, P=0.93, η^2^_P_=0.001) groups presented higher values compared to the Ob group (P<0.05) in the final MPET, as well as when compared to each other in the initial and final MPET (P<0.05). An effect of HIIT was observed on the final MPET of the speed, distance, and time parameters (ObHsHIIT>ObHs; (speed: F_1,25_=83.24, P<0.001, η^2^_P_=0.77; distance: F_1,25_=83.69, P<0.0001, η^2^_P_=0.77; and time: F_1,25_=43.44, P<0.0001, η^2^_P_=0.63). No statistical differences were observed in lactate analyses (HIIT effect: F_1,25_=0.08, P=0.93, η^2^_P_=0.0001; Hs effect: F_1,25_=0.44, P=0.51, η^2^_P_=0.02; and HIIT × Hs effect: F_1,25_=0.03, P=0.86, η^2^_P_=0.001).

**Figure 2:**
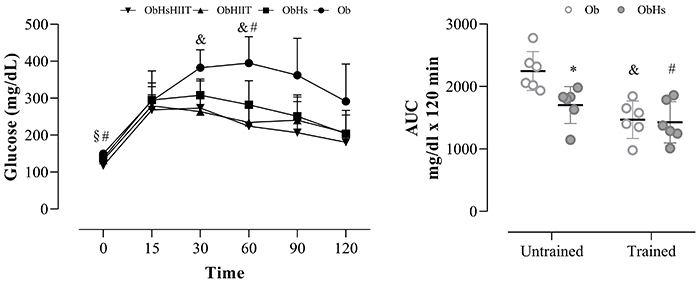
Effect of the interaction of *Hibiscus sabdariffa* and high-intensity interval training on blood glucose and area under the curve (AUC). Groups C: control (n=6); Ob: obese (n=6); ObHs: obese + *Hibiscus sabdariffa* (n=6); ObHIIT: obese + high-intensity interval training (n=6); ObHsHIIT: obese + *Hibiscus sabdariffa* + high-intensity interval training (n=6). Data are reported as means±SD. Two-way repeated measures ANOVA for independent samples, complemented with Tukey's *post hoc* test. AUC: two-way ANOVA for independent samples, complemented with Tukey's *post hoc* test. *P<0.05 Ob *vs* ObHs; ^&^P<0.05 Ob *vs* ObHIIT; ^#^P<0.05 Ob *vs* ObHsHIIT; ^§^P<0.05 ObHs *vs* ObHsHIIT.

Figure 2 shows the data related to the glycemic curve obtained from the glucose tolerance test. The glycemic curve between groups C and Ob showed significant results, being elevated in the Ob group at baseline (C: 115± 10 *vs* Ob: 149±9 mg/dL, P<0.05) and after 30 (C: 213±26 *vs* Ob: 382±43 mg/dL, P<0.05), 60 (C: 175±9 *vs* Ob: 395±65 mg/dL, P<0.05), and 90 min (C: 151±24 *vs* Ob: 362±91 mg/dL, P<0.05) after glucose administration (Time effect: F_1,25_=15,58, P=0.0002, η^2^_P_=0.76; Obesity Effect: F_1,25_=76.42, P<0.0001, η^2^_P=_0.94; and Time × Obesity effect: F_1,25_=7.09, P=0.0001, η^2^_P_=0.59). Additionally, the AUC between groups C and Ob presented significant results, being elevated in the Ob group (C: 1130±79 mg/dL/min *vs* Ob: 2246±285 mg/dL/min (P<0.0001, effect size 5.34).

Regarding the glycemic curve data of treated groups (Figure 2), at baseline, the ObHsHIIT group presented lower glycemia values compared to the Ob and ObHs groups. After 30 min of glucose administration, the ObHIIT group showed a reduction of 31% compared to the Ob group. At 60 min, ObHIIT and ObHsHIIT groups presented the lowest glycemic values compared to the Ob group, a reduction of 40.6 and 43.2% respectively (Time effect: F=60.67, P=0.0001, η^2^_P_=0.92; Group Effect: F=5.44, P=0.01, η^2^_P_=0.52; and Time × Group effect: F=2.14, P=0.02, η^2^_P_=0.30). There were no statistical differences in other moments among the groups. It is important to note that glucose returned to baseline values after 120 min in all groups. Concerning AUC, the ObHs, ObHIIT, and ObHSHIIT groups presented lower values compared to the Ob group (a reduction of 24.2, 34.6, and 36.4% respectively).

**Figure 3:**
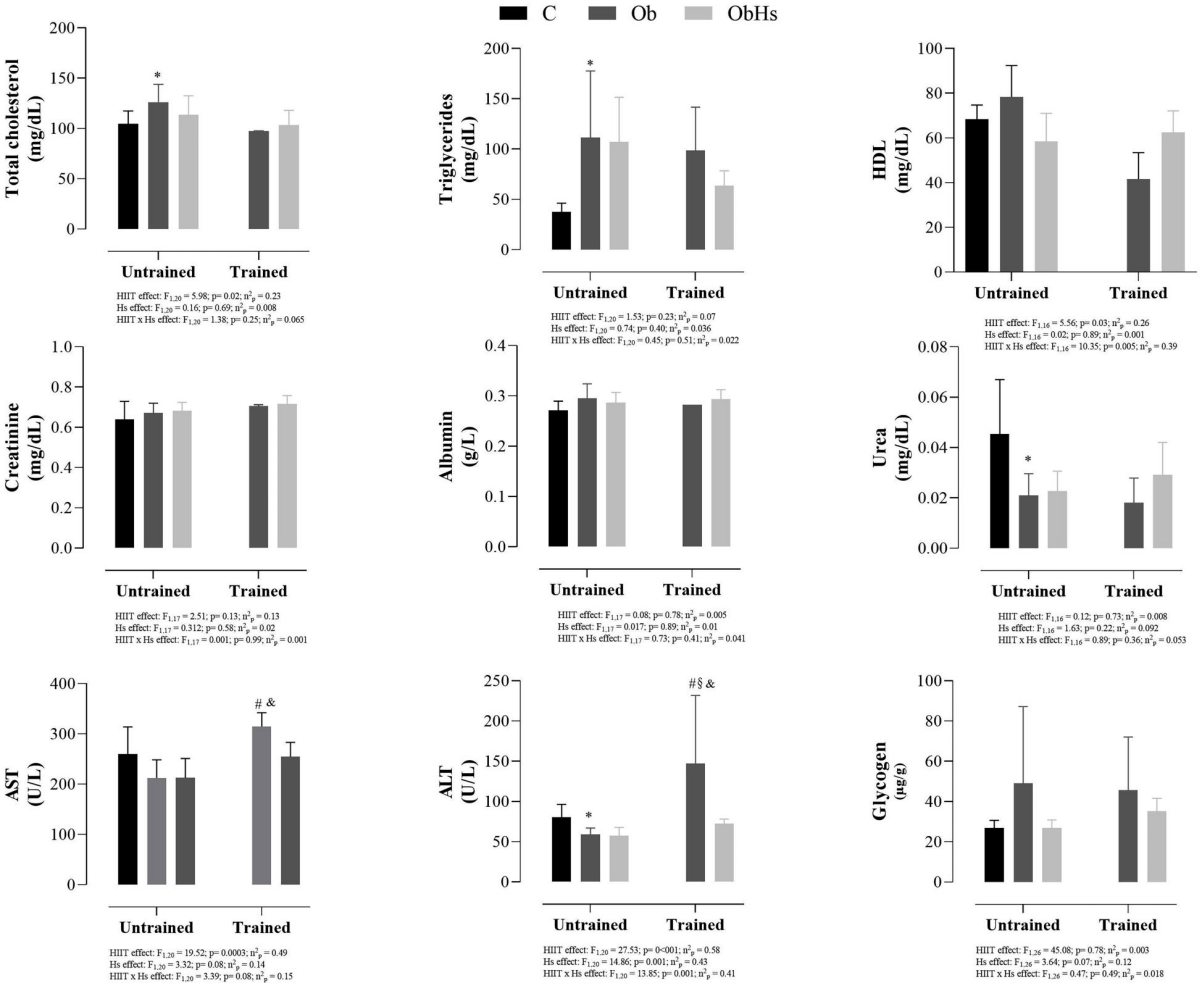
Biochemical and liver glycogen parameters of the experimental groups. AST: aspartate aminotransferase; ALT: alkaline phosphatase. Groups C: control (n=7); Ob: obese (n=8); ObHs: obese + *Hibiscus sabdariffa* (n=7); ObHIIT: obese + high-intensity interval training (n=2); ObHsHIIT: obese + *Hibiscus sabdariffa* + high-intensity interval training (n=7). Data are reported as means±SD. Student’s *t*-test for independent samples (C and Ob groups). *P<0.05 C *vs* Ob; Two-way ANOVA for independent samples in obese groups (Ob, ObHS, ObHIIT, and ObHsHITT), complemented with Tukey's *post hoc* test. ^#^P<0.05 compared to other groups; ^&^P<0.05 ObHs *vs* ObHIIT; ^§^P<0.05 ObHIIT *vs* ObHsHIIT.

Figure 3 shows biochemical and liver glycogen data of the experimental groups. Regarding the triglycerides (P=0.01, effect size 1.56) and total cholesterol (P=0.02, effect size 1.4), the Ob group presented significantly higher values compared to the C group (P<0.05). Nevertheless, urea (P=0.03, effect size 1.41) and ALT (P=0.005, effect size 1.72) were reduced in the Ob group compared to the C group. The ObHIIT group presented significantly higher values of AST compared to the Ob and ObHs groups. This elevation was of 48.1 and 47.9%. In addition, the results showed that ALT was elevated in the ObHIIT group in relation to Ob, ObHs, and ObHsHIIT groups, representing an increase of 149, 158, and 102%, respectively. In the trained groups (ObHIIT *vs* ObHsHIIT), a hepatoprotective effect of Hs was observed, with decreased ALT values in the ObHsHIIT group. No statistical differences were observed in the other parameters.

## Discussion

The objective of this study was to investigate the association between the administration of Hs extract combined with HIIT and their protective role on metabolic adaptations and damage in lipid metabolism induced by obesity. The main findings of this study were that the Hs and HIIT association promoted a reduction in food parameters and body adiposity, as well as an improvement in physical performance, glycemic profile, and a hepatoprotective effect, but without alterations in lipid metabolism. In addition, we found an isolated and beneficial effect of HIIT on body fat in obese rats with an improvement in adiposity.

The hypercaloric diet used in the current study was effective in promoting obesity, indicated by the difference in FBW and BW gain, epididymal, visceral, and retroperitoneal fat deposits, total fat, and adiposity index between groups C and Ob (Table 2). In line with our findings, the study by dos Santos et al. (23) shows that hypercaloric diets induce and maintain obesity in experimental models due to their high lipogenic potential, promoting obesity observed by the difference in body composition and adiposity between the control and obese groups.

Different strategies have been described in the literature as adjuvant tools in the treatment and management of obesity. Among the possible new pharmacological treatments are the use of bioactive substances isolated from *Hibiscus sabdariffa* in association with other non-pharmacological tools (24). In this sense, HIIT has been recognized in the literature as a very important non-pharmacological tool for managing obesity, showing promising results in this condition and associated diseases (19,23).

In the current study, one of the main findings was the isolated effect of HIIT on the body fat of obese rats. This factor was responsible for the main effect in the association with Hs, which potentiated the reduction of adiposity in obesity. Studies have shown that HIIT can effectively reduce body fat and fat pads as observed in the current study (25,26). Lipolysis in adipocytes can be activated by catecholamine release by the sympathetic nervous system and adrenal glands through β3-adrenergic receptors, which are the major regulatory pathway of adipose catabolism. HIIT breaks down stored fat into triglyceride, resulting in glycerol and non-esterified fatty acid, and consequently increasing the availability of fatty acids to be used as an energy source during exercise (27). In addition, as the main substrate during exercise is glycogen, the mechanism by which HIIT can reduce fat is generally suspected to be related to the ‘post-exercise' changes in fat catabolism (27). Therefore, these findings showed the beneficial effect of HIIT on obesity.

Previous data from our study group demonstrated that Hs extract reduced food consumption, body adiposity, and peripheral insulin resistance in obese rats induced by a high-fat diet (28). Other studies such as that by Boix-Castejón et al. (29) showed that overweight individuals who ingested 325 mg Hs capsules daily for 8 weeks had better body weight control and reduced food intake, which is in line with the findings of the present study.

Thus, one of the possible explanations for the anti-obesogenic effect of Hs is through modulation of appetite biomarkers leading to better control and reduction of body mass (28-30). Several studies suggest that the reduction of body adiposity by Hs is related to bioactive compounds, such as polyphenols, flavonoids, and anthocyanins. Hs promotes the activation of AMPK and the inhibition of SREBP-1 expression, which is the main transcription factor that intervenes in the activation of lipogenesis; thus, Hs extract can regulate lipid homeostasis through the inhibition of SREBP-1c and PPAR-γ, stimulating lipolytic and cardioprotective signaling pathways. Data from systematic reviews showed that Hs extracts inhibited NFk-B expression, which is responsible for the activation of cytokines such as TNF-α, which promotes mitochondrial dysfunction, causing lipid peroxidation instead of lipid oxidation, thus increasing free radicals and causing damage to hepatocytes. These antioxidant and metabolic effects show that Hs can be a potential substrate for the attenuation and improvement of mitochondrial oxidation and hepatoprotection (30,31). Nevertheless, it is important to note that in the current study the isolated effect of Hs in adiposity was not observed, but only when associated to HIIT, causing lower caloric intake, better feed efficiency, lower body weight gain and final BW, and reduced epididymal, visceral, and retroperitoneal fat pad deposits, total body fat, and adiposity index (Table 2).

The literature indicates that HIIT is also effective in reversing risk factors associated with obesity. Experimental studies using models of obesity induced by a high-fat diet with a HIIT treatment protocol have shown an improved physical performance and cardiorespiratory parameters, reaching higher speed, longer distance, and shorter time (20,32). In a similar way, our findings demonstrated better physical performance with improvements in distance, speed, and time parameters in the trained groups (ObHIIT and ObHsHIIT). HIIT promotes improvement in physical conditioning associated with fast adaptations in the cardiovascular system (23,28), as evidenced by the improvement of the initial and final MPET parameters.

A study carried out by Snook et al. (33) using obese Wistar rats on a high-fat diet (60% kcal from fat) and HIIT for 15 min/day, 5×/week for 8 weeks showed an improvement in glucose metabolism with a lower glycemic curve among trained obese groups, as well as a reduction in insulin resistance and body weight gain, which was related to increased energy expenditure. Similarly, another investigation demonstrated that HIIT (85-100% of VO_2_max) for 8 weeks promoted an improvement in glucose tolerance in rats with obesity induced by a high-fat diet (34). Machado et al. (35) evaluated the effect of different training duration and intensity in rats submitted to a high-calorie diet for 12 weeks. The authors found that the percentage of body fat reduction is dependent on the duration and intensity of the exercise. However, all training levels were effective in reducing insulin resistance.

One possible explanation for obesity reduction induced by the association of Hs and HIIT is the effect of Hs on appetite biomarkers and on the inhibition of enzymes of lipogenesis pathways, while HIIT promotes physiological adaptations by stimulating lipolysis pathways to provide energy during and after exercise sessions, since O_2_ consumption does not immediately return to resting levels, contributing to the reduction in body adiposity (24,30).

Hs and HIIT, alone or combined, were effective in improving the AUC and the glycemic curve in the glucose tolerance test in the experimental groups. Similarly, in the study by Mendes et al. (36), it was observed that HIIT reduced fasting glucose levels, reduced glucose intolerance, and improved insulin tolerance. The authors suggest that one of the possible mechanisms by which HIIT promotes this effect is by increasing the expression and translocation of glucose transporter (GLUT)-2 and GLUT-4, as well as improving insulin resistance (32).

Several molecular mechanisms have been implicated in the improvement of glycemic control induced by physical training and Hs. Regarding physical training, experimental evidence has shown that muscle contraction increases glucose uptake, via the protein kinase (AMPK) pathway, which is activated by adenosine monophosphate (AMP), promoting glucose transport via GLUT-4 to the plasma membrane independently of insulin. Exercise also increases the expression of GLUT-4, reduces oxidative stress, and improves insulin sensitivity (37).

Regarding the effects of Hs on glucose, the bioactive compounds (phenolics and anthocyanins) present in the Hs extract stimulate the expression of the insulin receptor via nuclear factors, increasing the sensitivity of cells to insulin and promoting the expression and translocation of GLUT-4 in the cell membrane, contributing to the maintenance of glycemic levels and energy generation (29,30). Therefore, the improvement in glycemic parameters induced by the two therapies, alone or combined, in obese rats observed in our study may be due to the better glucose transport capacity via GLUT-4 and the improvement in antioxidant capacity promoted by Hs supplementation. Nevertheless, this pathway was not evaluated in the current study.

Regarding biochemical parameters and lipid metabolism, the Hs-HIIT combination was not effective in improving the metabolic, lipid, and hepatic profile of the experimental groups. However, Hs-HIIT showed a hepatoprotective effect, decreasing ALT levels in the ObHsHIIT group compared to the HIIT group. Our findings were in agreement with those of Ezzat et al. (38) who found that the Hs extract was reduced the levels of AST, ALT, and hepatic malondialdehyde by 37, 96, and 42% respectively. The authors reported that Hs was able to decrease hepatic inflammatory markers, including TNF-α, IL-6, and interferon gamma (INF-γ). However, these effects were not observed in the current study.

One important aspect that did not support our initial hypothesis was that HIIT alone did not promote an elevation in HDL and a reduction in total cholesterol, TG, LDL, AST, and ALT levels. Differing from our findings, studies in the literature indicate that obese rats submitted to a HIIT protocol for 8 weeks, 5 days/week, (39,40) showed a reduction in metabolic changes and liver lipids compared to the obese group.

We recognize some limitations in the current study: 1) It was not possible to carry out hormonal analysis of insulin, glucagon, ghrelin, and leptin, which are important for discussing metabolic adaptation in obesity and the role of HIIT and *H. abdariffa*; 2) As several obesity-induced changes occur in the liver, morphological analysis of a cross-sectional area and fat droplets of the liver could indicate the role of the two non-pharmacological strategies as prevention and/or treatment tools.

### Conclusion

Further studies with molecular approaches are necessary to elucidate the pathways and interactions of the combined treatments in obesity and lipid metabolism. The association of the two therapies promoted a reduction in food consumption and body adiposity in obesity, as well as an improvement in physical performance and glycemic profile, but without changes in lipid metabolism. Nevertheless, the effects of Hs were more pronounced when associated with HIIT. Therefore, the isolated or associated use of HIIT and Hs showed promising results in the management of obesity.
